# β-catenin promotes the type I IFN synthesis and the IFN-dependent signaling response but is suppressed by influenza A virus-induced RIG-I/NF-κB signaling

**DOI:** 10.1186/1478-811X-12-29

**Published:** 2014-04-26

**Authors:** Andrea Hillesheim, Carolin Nordhoff, Yvonne Boergeling, Stephan Ludwig, Viktor Wixler

**Affiliations:** 1 Institute of Molecular Virology (IMV), Centre for Molecular Biology of Inflammation (ZMBE), Westfaelische Wilhelms-University Muenster, Von-Esmarch-Str. 56, 48149 Muenster, Germany

**Keywords:** Influenza A virus, Innate immune response, β-catenin, γ-catenin, Interferon-β, Interferon-stimulated genes, β-catenin-dependent target genes

## Abstract

**Background:**

The replication cycle of most pathogens, including influenza viruses, is perfectly adapted to the metabolism and signal transduction pathways of host cells. After infection, influenza viruses activate several cellular signaling cascades that support their propagation but suppress those that interfere with viral replication. Accumulation of viral RNA plays thereby a central role. Its sensing by the pattern recognition receptors of the host cells leads to the activation of several signal transduction waves that result in induction of genes, responsible for the cellular innate immune response. Type I interferon (IFN) genes and interferon-stimulated genes (ISG) coding for antiviral-acting proteins, such as MxA, OAS-1 or PKR, are primary targets of these signaling cascades. β- and γ-catenin are closely related armadillo repeat-containing proteins with dual roles. At the cell membrane they serve as adapter molecules linking cell-cell contacts to microfilaments. In the cytosol and nucleus, the proteins form a transcriptional complex with the lymphoid enhancer factor/T-cell factor (LEF/TCF), regulating the transcription of many genes, thereby controlling different cellular functions such as cell cycle progression and differentiation.

**Results:**

In this study, we demonstrate that β- and γ-catenin are important regulators of the innate cellular immune response to influenza A virus (IAV) infections. They inhibit viral replication in lung epithelial cells by enhancing the virus-dependent induction of the *IFNB1* gene and interferon-stimulated genes. Simultaneously, the prolonged infection counteracts the antiviral effect of β- and γ-catenin. Influenza viruses suppress β-catenin-dependent transcription by misusing the RIG-I/NF-κB signaling cascade that is induced in the course of infection by viral RNA.

**Conclusion:**

We identified β- and γ-catenin as novel antiviral-acting proteins. While these factors support the induction of common target genes of the cellular innate immune response, their functional activity is suppressed by pathogen evasion.

## Background

Influenza A viruses (IAV) represent worldwide circulating pathogens that cause seasonal epidemics but also occasionally lead to severe pandemic outbreaks. These viruses belong to the family of *orthomyxoviridae* and consist of a single-stranded RNA genome with negative orientation, which is organized in eight RNA segments. The RNA strands encode up to 14 viral proteins including structural and non-structural (NS) proteins [1-4]. Some of these, such as NS1 or PB1-F2, are adapted to prevent cellular and host immunity by manipulating multiple host signaling cascades [5-7]. Virus-infected cells generally respond to infection by induction of an innate immune response that is initiated by several cellular pattern recognition receptors (PRRs), which detect specialized pathogen-associated molecular pattern (PAMPs) molecules. In the case of IAV infections, the family of cytoplasmic retinoic acid-inducible gene-like (RIG-I) receptors are sensors for accumulating viral 5′-triphosphate RNA [8,9], resulting in the activation of the first line of defense, the type I interferon (IFN) response. This comprises the expression of IFN-α/β and the subsequent transcriptional activation of interferon-stimulated genes (ISG) [10].

Secreted IFN-β itself does not have direct antiviral action, but it induces in an auto- and paracrine manner the expression of antiviral-acting genes [10-12]. Binding of IFN-β to the type I interferon receptor (IFNAR1) activates the JAK/STAT signaling cascade. This results in formation of the IFN-stimulated gene factor 3 (ISGF3) protein complex consisting of the signal transducers and activators of transcription 1/2 (STAT1/2) and the interferon regulatory factor 9 (IRF9). This protein complex translocates into the nucleus and binds to IFN-stimulated response elements (ISRE) on the promoters of several ISGs [10], such as *myxovirus resistance gene a* (*MX1*) [13], *2-5-oligoadenylate synthetase* (*OAS*) [14] or *protein kinase R* (*EIF2AK2*) [15], thereby initiating their transcription. The translated proteins of these ISGs directly or indirectly interfere with virus replication and, thus, limit virus spread.

The highly conserved β-catenin protein is the vertebrate’s homologue of *Drosophila* armadillo. It consists of 781 amino acids, which form 12 so called armadillo repeats that are responsible for interactions with several proteins, such as cadherins, α-catenin, adenomatous polyposis coli (APC) or lymphoid enhancer factor/T-cell factor (LEF/TCF) [16-18]. In unstimulated cells, most β-catenin molecules function as adapter molecules at the cell membrane, linking cadherin receptors to the actin cytoskeleton. Simultaneously, a minor cytosolic pool of β-catenin acts upon association with LEF/TCF as a transcription factor. The relation between adhesional and transcriptional pools is dynamic and is regulated via phosphorylation of β-catenin at different amino acids at both the N- and the C-termini [19].

Most of the regulation of the β-catenin signaling cascade is mediated by the glycogen synthase kinase 3β (GSK-3β) and casein kinase 1α (CK1α) [20]. In unstimulated cells, they form a cytoplasmic protein degradation complex with axin, APC and the protein phosphatase 2A (PP2A). When bound to this complex, β-catenin is phosphorylated by the kinases at amino acids Ser33, Ser37, Thr41 and Ser45. The hyperphosphorylated β-catenin is then ubiquitinylated by the β-transducin repeat-containing protein (β-TrCP) and subsequently degraded by the 26S proteasome [20,21]. Activation of the Wnt signaling cascade leads to the dissociation of the degradation complex [22,23] and inactivation of the GSK-3β via phosphorylation at Ser9 [24]. Consequently, the non-phosphorylated β-catenin is released and interacts with LEF/TCF, forming a transcriptional complex that induces, together with other co-factors like CBP/p300, the expression of many genes. The most prominent of these are the cell cycle inducing cyclin D1 [25,26] and the transcription factor c-Myc [27]. Besides Wnt factors [24], stimulation of cells with insulin [28], EGF [29] or inducers of the PI3K [30] also might result in inactivation of GSK-3β and transcriptional activation of β-catenin.

The γ-catenin molecule, also known as plakoglobin, is a very close homologue of β-catenin. It is also able to exert a dual role within the cell and act as an adapter molecule [31] and, in concert with LEF/TCF, as a transcription factor, regulating the expression of common β-catenin target genes, although to a lesser extent than β-catenin itself [32,33].

During IAV infection, several cellular signaling cascades are activated that may support or inhibit viral replication. The PI3K/Akt signaling axis is a prominent pathway with a dual action with respect to influenza viruses [34]. Activation of this pathway also results in the phosphorylation of GSK-3β at Ser9 [30], suggesting an accumulation and activation of β-catenin during IAV infection.

In this study, we demonstrate that β-catenin and its closely related homolog γ-catenin are important regulators of the innate cellular immune response to IAV infections. They inhibit virus replication in lung epithelial cells by enhancing the virus-dependent induction of the type I IFN system. However, the transcriptional activity of β-catenin is simultaneously inhibited upon viral infection by the RIG-I signaling cascade that is induced by influenza viral RNA.

## Results

### Accumulation of β- and γ-catenin decreases influenza A virus propagation

**Figure 1:**
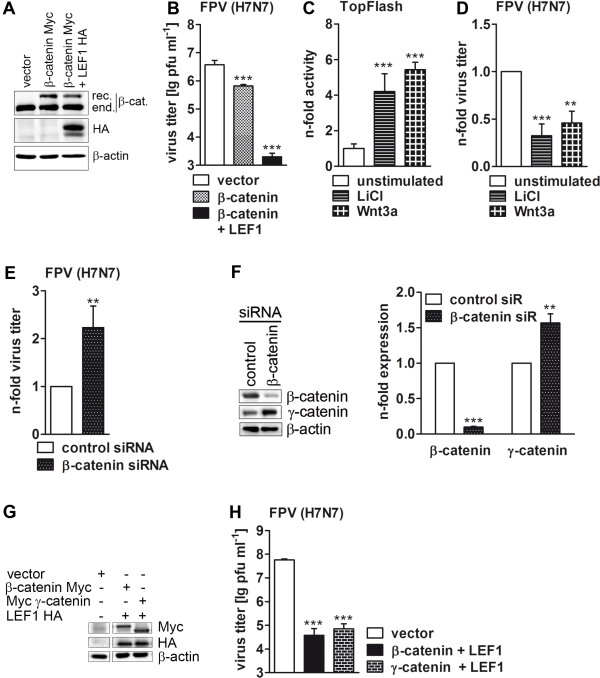
**Accumulation of cellular β- and γ-catenin reduces influenza A virus progeny. (A and B)** A549 cells were transfected with the indicated plasmids (1 μg each but 2 μg per 6-well dish) for 24 h and subsequently infected with FPV (MOI = 0.01) for an additional 24 h. Transfection efficiency was verified by Western blotting. Equal protein loads were verified by β-actin immunoblotting **(A)**. Virus propagation was analyzed by standard plaque titration assay **(B)**. Representative images of three independent experiments are depicted. **(C)** A549 cells were stimulated with 20 mM LiCl or 100 mg/ml recombinant Wnt3a for 20 h and the induction of β-catenin signaling was verified by measuring the activity of the transfected TopFlash reporter gene. Luciferase activity of unstimulated cells was taken as unity. **(D)** A549 cells were prestimulated with 20 mM LiCl or 100 mg/ml Wnt3a for 3 h and 16 h, respectively, and infected with FPV (MOI = 0.01) for 24 h. Virus propagation was analyzed by standard plaque titration assay and the titer of unstimulated cells was arbitrarily set to 1. One representative experiment out of three is shown. **(E and F)** The endogenous β-catenin of SW480 cells was knocked down by specific siRNA. At 24 h post transfection, cells were infected with FPV (MOI = 0.001) for an additional 24 h, and viral titers **(E)** as well as knockdown efficiency **(F)** were determined. The β-actin immunoblot served as loading control. Protein scores of β- and γ-catenin presented in the right panel of **(F)** were estimated densitometrically as the relative intensity of the respective protein bands to the loading control. Mean values of control cells were taken as unity. **(G and H)** A549 cells were transfected with the indicated plasmids for 24 h and subsequently infected and analyzed as in **(A and B)**.

To elucidate whether accumulation of cellular β-catenin influences viral replication, we overexpressed the protein in human lung epithelial A549 cells by plasmid transfection prior to IAV infection and subsequently analyzed the efficiency of viral propagation. To ensure that the recombinant β-catenin is not degraded by the proteasome, the phosphorylation-refractory β-catenin substitution mutant S33A [35] was used. A549 cells, transfected with empty vector, served as control and the expression efficiency of the transgene was monitored by Western blot analysis (Figure 1A). As shown in Figure 1B overexpression of the transcriptionally active β-catenin significantly impaired the replication of avian FPV (A/FPV/Bratislava/79 (H7N7)) influenza A viruses compared to vector control-transfected cells. Because β-catenin exerts its gene expression function in concert with the transcription factor LEF1, the effect of their co-expression was analyzed as well and indeed LEF1 boosted the antiviral effect of β-catenin on FPV replication dramatically (Figures 1A and B).

To further explore whether the accumulation of endogenous β-catenin also inhibits virus replication, the intracellular pool of β-catenin was augmented by stimulation of A549 cells with either lithium chloride (LiCl) or the glycoprotein Wnt3a. LiCl is a commonly used drug for treatment of bipolar disorders with clinical relevance for more than 50 years and is known to inhibit the GSK-3α and β isoforms [36]. Wnt3a is a known activator of the canonical Wnt signaling cascade. To confirm that both stimuli lead to accumulation of transcriptionally active β-catenin, A549 cells were transfected with a β-catenin/LEF1-dependent TopFlash reporter construct harboring LEF/TCF binding sites upstream of the thymidine kinase minimal promoter [35] for 24 h and stimulated with LiCl or Wnt3a for further 20 h. Measuring the luciferase activity of the reporter gene product showed that the TopFlash-specific promoter activity was indeed significantly increased by both stimuli (Figure 1C). Next, A549 cells were prestimulated with LiCl for 3 h or with Wnt3a for 16 h and subsequently infected with avian FPV in the presence of the stimuli for an additional 24 h. Both LiCl and Wnt3a stimulation of A549 cells reduced virus propagation by approximately 50% (Figure 1D).

To further confirm the antiviral properties of endogenous β-catenin, its amount was downregulated by an siRNA approach. For this purpose, SW480 colon carcinoma cells were used. They carry mutations in the *APC* gene that prevent formation of the protein degradation complex and result in accumulation of intracellular β-catenin [37-39]. As expected, the RNAi-based downregulation of β-catenin was associated with an increase in viral replication (Figure 1E). Although the differences in viral titers were significant compared to cells transfected with control siRNA, the effect was, nonetheless, much less than expected. This might be due to the simultaneous increase in γ-catenin expression in colon carcinoma cells that occurs upon β-catenin knockdown (Figure 1F), a fact that has been previously described for hepatocytes [40]. This suggests that γ-catenin also might be involved in reducing viral propagation. And indeed, we could show that this protein also possesses antiviral potential, since in cells overexpressing γ-catenin along with LEF1 (Figure 1G) a significant decrease in viral replication compared to cells overexpressing control vectors was observed (Figure 1H). Analysis of viral replication in cells with downregulated expression of both β- and γ-catenin was not possible, as RNAi-knockdown of both proteins led to cell death shortly after virus infection (data not shown).

**Figure 2:**
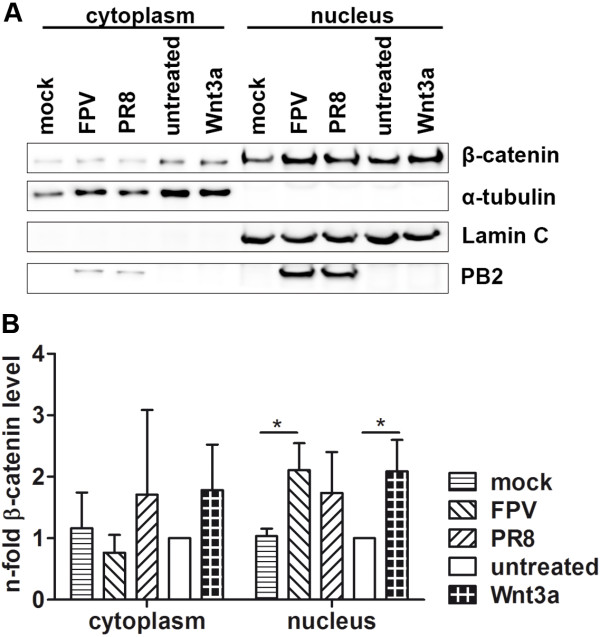
**IAV infection induces nuclear accumulation of β-catenin molecules.** HEK293 cells were stimulated with 100 mg/ml Wnt3a or infected with either FPV or PR8 (MOI = 5) for 6 h. Subsequently, cell lysates were fractionated and the cytosolic and nuclear fractions were analyzed by SDS-PAGE and Western blotting for β-catenin levels. **(A)** Immunoblotting of α-tubulin and Lamin C served as internal loading controls for cytosolic and nuclear fractions, respectively. The infection was verified by expression of viral PB2 protein. One representative Western Blot out of three is depicted. **(B)** Relative β-catenin protein scores of Western blots are presented. They were estimated densitometrically as the relative intensity of the appropriate protein band to the loading control and normalized to the cytoplasmic or nuclear fraction of untreated cells, respectively. Values are means of three independently repeated experiments.

As inactivation of GSK-3β is one of the causes for cellular accumulation of endogenous transcriptionally active β-catenin [41], we monitored the phosphorylation status of GSK-3β at Ser9 by Western blotting after infection of cells with the avian FPV (A/FPV/Bratislava/79 (H7N7)) or human PR8 (A/Puerto Rico/8/34 (H1N1)) influenza virus strains (Additional file 1: Figures S1A and B). The phosphorylation of Ser9 was detectable after infection with both IAV strains from 6 h p.i. and lasted at least up to 10 h p.i., thus, showing that infection of cells with influenza viruses results in inactivation of GSK-3β and assuming an accumulation of endogenous β-catenin within the cell. Indeed, analysis of cytosolic and nuclear β-catenin levels confirmed this and showed that infection of cells with influenza viruses resulted, similar to Wnt3a stimulation, in accumulation of cellular β-catenin protein, mostly in the nucleus (Figure 2).

Taken together, these data show that accumulation of transcriptionally active β-catenin or γ-catenin impairs IAV propagation.

### Catenins regulate interferon-β induction

**Figure 3:**
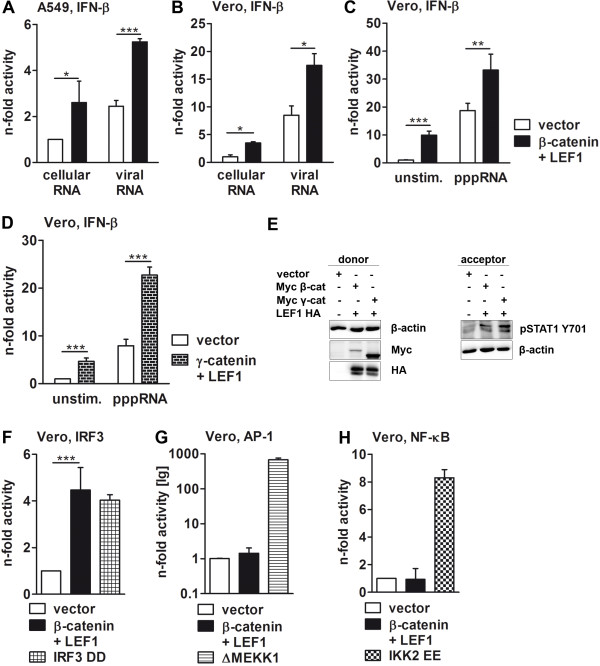
**Regulation of the IFN-β promoter by catenins and LEF1. (A-D)** A549 or Vero cells were transfected with IFN-β luciferase gene reporter plasmid (indicated above the images) along with plasmids specified in column legends for 24 h and either left untreated or stimulated (by transfection) for 5 h with 500 ng of cellular RNA (isolated from uninfected A549 cells), viral RNA (isolated from PR8-infected A549 cells) or 1 μg of 5′-triphosphate modified RNA (pppRNA) per well. Luciferase activity of unstimulated or cellular RNA-stimulated and empty vector-transfected cells was taken as unity. **(E)** A549 cells were transfected with indicated plasmids for 24 h and conditioned media (donor cells) were harvested before the cells were lysed. The RIPA cell lysates were then analyzed for the efficacy of transfection by Western blotting (left panel). The conditioned media were applied to freshly plated A549 cells (acceptor cells) for 15 min and cells stimulated with conditioned media were then analyzed for phosphorylation of STAT1 at Y701 (right panel). Immunoblotting of β-actin served as loading controls. **(F-H)** Vero cells were transfected with luciferase reporter plasmids containing either the IRF3- **(F)**, AP-1- **(G)** or NF-κB- **(H)** dependent binding region of the IFN-β enhanceosome along with plasmids indicated in column legends. The activity of reporter genes was determined 30 h after transfection. Luciferase activity of empty vector-transfected cells was always taken as unity. Mean values from three independent experiments are depicted in **A**, **D**, **F**, **G** and **H**, whereas **B**, **C** and **E** show representative images of three independent experiments.

The innate immune response of virus-infected cells is primarily governed by rapid transcription, translation and secretion of IFN-β, which is triggered by the accumulation of newly produced viral RNA. Subsequently, secreted IFN-β acts in an auto- and paracrine fashion to induce the expression of proteins coded by interferon-stimulated genes that, in turn, suppress viral propagation [10]. Thus, we wondered whether accumulation of intracellular β-catenin is involved in regulating the IFN-β induction and thereby executes its antiviral potential. To explore this, A549 cells were transiently co-transfected with β-catenin and LEF1 together with a luciferase reporter gene construct driven by the IFN-β enhanceosome, a promoter element that contains all principal transcription factor binding sites of the IFN-β promoter. Twenty-four hours post transfection, the cells were stimulated for an additional 5 h with total RNA isolated from non-infected (cellular RNA) or influenza A virus-infected (viral RNA) A549 cells. The latter RNA sample is mimicking the release of viral RNA upon IAV infection. Overexpression of the β-catenin/LEF1 complex significantly increased the IFN-β enhanceosome activity (Figure 3A), suggesting that β-catenin and LEF1 strongly support the transcription of the *IFNB1* gene in lung epithelial cells. Interestingly, the β-catenin/LEF1-mediated induction of IFN-β transcription could be measured independently of whether cells were stimulated with viral RNA or not.

Furthermore, the capability of β-catenin and LEF1 to stimulate IFN-β induction was cell type-independent, as overexpression of these proteins in Vero cells showed a similar effect (Figure 3B). These cells are deficient in the production of type I IFNs [42] and, thus, represent a good tool for investigation of IFN response independently of the IFN synthesis. Also here, expression of β-catenin and LEF1 enhanced the activity of the IFN-β promoter, regardless of whether the cells were unstimulated or stimulated with RNA from virally-infected cells or synthetically 5′-triphosphate modified RNA (pppRNA) that represents the typical structure of IAV vRNA (Figures 3B and C).

As γ-catenin exhibits similar antiviral activity as β-catenin (Figure 1H), we questioned whether γ-catenin is also able to enhance IFN-β enhanceosome activity. Figure 3D clearly demonstrates that, similar to β-catenin, γ-catenin significantly enhanced together with LEF1 the promoter activity, in unstimulated as well as in pppRNA-stimulated cells.

IFN-β is a secreted cytokine affecting cells in an autocrine and paracrine manner by binding to the type I IFN receptor. This induces a signaling cascade leading to the phosphorylation of the transcription factor signal transducer and activator of transcription 1 (STAT1) and expression of ISGs [10]. To analyze whether the enhanced IFN-β promoter activity induced by overexpression of β-catenin or γ-catenin together with LEF was also translated into increased expression and secretion of functional IFN-β molecules, freshly plated A549 cells were treated with supernatants of either control or catenin and LEF transfected cells and the phosphorylation of STAT1 at tyrosine 701 was monitored by Western blotting. The results of Figure 3E (right panel) clearly show that phosphorylation of STAT1 in A549 cells was increased after stimulation with supernatants from catenin and LEF1 overexpressing cells (Figure 3E, left panel), which is in accordance with the enhanced IFN-β promoter activity.

The activity of the IFN-β enhanceosome is dependent on members of three different transcription factor families, the interferon regulatory factors (IRFs), activator proteins (AP) and the nuclear factor kappa-light-chain-enhancers of activated B-cells (NF-кB) that bind to the positive regulatory domains (PRD) in the promoter region of the *IFNB1* gene [10]. To decipher which of these three binding sites is supported by the β-catenin/LEF1 complex, reporter gene assays with constructs harboring sites for either IRF3, AP-1 or NF-кB were performed. Transfection of Vero cells with these constructs demonstrated that only the IRF3 (Figure 3F), but not AP-1 (Figure 3G) or NF-кB (Figure 3H) responsive elements were efficiently activated by overexpressed β-catenin and LEF1. The inability of β-catenin and LEF1 to induce AP-1 and NF-кB PRD activation was not due to a functional inactivity of the used constructs, as expression of positive controls like constitutively active MEKK1 (mitogen-activated protein/extracellular signal-regulated kinase kinase kinase 1) or the inhibitor of kappa B kinase 2 (IKK2), respectively, were able to induce expression of the luciferase enzyme driven by these promoters (Figures 3G and H, right columns). Hence, these data demonstrate that both β-catenin and γ-catenin are efficient stimulators of the IFN-β promoter and that this activity is mainly executed via the IRF3-specific region.

### β-catenin acts in concert with the p300 transcription co-factor and binds the IFN-β promoter

It is known that the transcription factor IRF3 is the main driver of IFN-β expression upon IAV infection. Accumulating viral RNA binds to and activates the intracellular RIG-I receptor, which signals via the adaptor protein MAVS (mitochondrial antiviral-signaling protein) to kinases such as TBK-1 (TANK-binding kinase 1) and IKKϵ (I-kappa-B kinase epsilon) which in turn phosphorylate IRF3. Phosphorylated IRF3 molecules form active dimers that migrate into the nucleus and initiate, together with CBP/p300, IFN-β transcription [43]. Furthermore, in independent experiments it has been shown that β-catenin directly interacts with IRF3 [44] and with CBP/p300 as well [45]. However, it is conceivable that the β-catenin/LEF1 protein complex supports the IRF3-mediated transcription either by augmenting IRF3 phosphorylation and, hence, dimerization in the cytosol or directly as transcription factor in the nucleus. Although our experiments with cellular RNA-stimulated cells already suggested that β-catenin/LEF1 does not act in the IFN-β activation pathway provoked by viral RNA, this was further affirmed via comparison of the dimerization capacity of IRF3 on stimulation with viral RNA in the absence or presence of β-catenin and LEF1 (Additional file 1: Figure S2). Thus, the β-catenin/LEF1 protein complex does not support the transcriptional activity of IRF3 via modulation of IRF3 dimerization but rather through enhancing its transcriptional activity in the nucleus.

**Figure 4:**
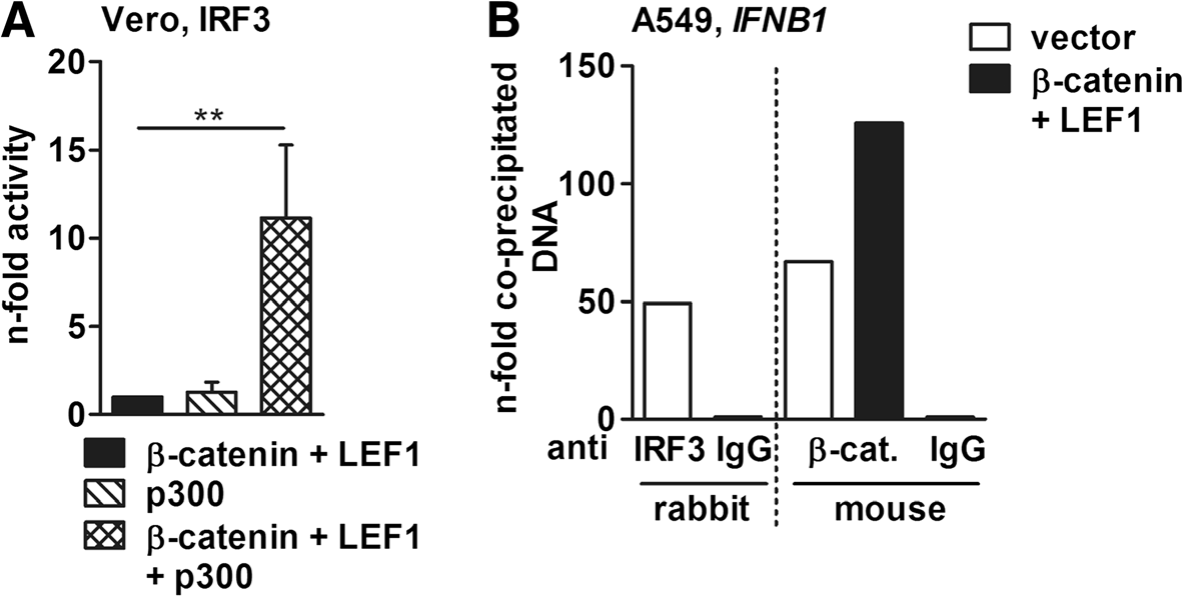
**β-catenin and LEF1 regulate IFN-β promoter activity via IRF-PRD. (A)** Vero cells were transfected with the reporter gene plasmid containing the IRF3-responsive elements of the IFN-β enhanceosome along with indicated plasmids, and the reporter gene activity was measured 30 h later. The luciferase activity of β-catenin and LEF1-transfected cells was arbitrarily taken as unity. **(B)** A549 cells were transfected with empty vector or plasmids coding for β-catenin and LEF1 for 30 h, and the interaction of cellular proteins with the DNA was analyzed by ChIP assays. The amount of amplified DNA in IRF3- and β-catenin-specific immunoprecipitates was quantified by qRT-PCR using primers specific for the promoter region of the *IFNB1* gene. Values represent n-folds of IgG controls. One of three independently repeated experiments is depicted as representative.

It is known that phosphorylated IRF3 dimers recruit the acetyltransferase CBP/p300 to the IFN-β promoter [43] and, recently, Yang et al. showed that β-catenin also promotes the recruitment of p300 to the IFN-β promoter via direct interaction with IRF3 [44]. These data together with the reporter gene assay and IRF3 gel shift results suggest that the β-catenin/LEF1 complex modulates the transcriptional activity of IRF3 in concert with the general co-activator CBP/p300. To verify this assumption, the IRF3-dependent luciferase reporter gene activity in cells that overexpress only p300, β-catenin and LEF1 or all three proteins together were compared (Figure 4A). Overexpression of p300 alone already induced the IRF3-dependent promoter activity, but the extent was comparable to that of β-catenin and LEF1; however, when p300 was co-expressed with β-catenin and LEF1, the IRF3-promoting effect shown in Figure 3F was significantly enhanced (Figure 4A, right column).

To monitor whether the gain of promoter activity was due to binding of the β-catenin/LEF1 complex to the IFN-β enhanceosome, ChIP assays were performed (Figure 4B). As proof of principle, the endogenous IRF3 protein was precipitated with a specific antibody from control cells, transfected with vectors only, and the presence of IFN-β promoter DNA in the immunoprecipitate was analyzed by qRT-PCR using primers specific for the IFN-β promoter region. Importantly, IFN-β-specific signals could only be detected when the IRF3, but not the IgG-mediated immunoprecipitate was used as template in the qRT-PCR. Next, β-catenin was immunoprecipitated from vector or β-catenin and LEF1-transfected cells to demonstrate that β-catenin binds to the IFN-β promoter. Using the DNA present in these immunoprecipitates as templates for qRT-PCR confirmed the association of β-catenin with the IFN-β promoter region. Of note, both endogenous and recombinantly expressed β-catenin were able to bind the IFN-β promoter, however, an increased signal was measured in immunoprecipitates from β-catenin- and LEF1-transfected cells. No signal was detected when immunoprecipitations were performed with unspecific IgG antibodies.

Taken together, these results indicate that the β-catenin/LEF1 complex stimulates the IRF3-dependent transcription of IFN-β by interaction with the promoter DNA.

### Catenins potentiate ISG expression

**Figure 5:**
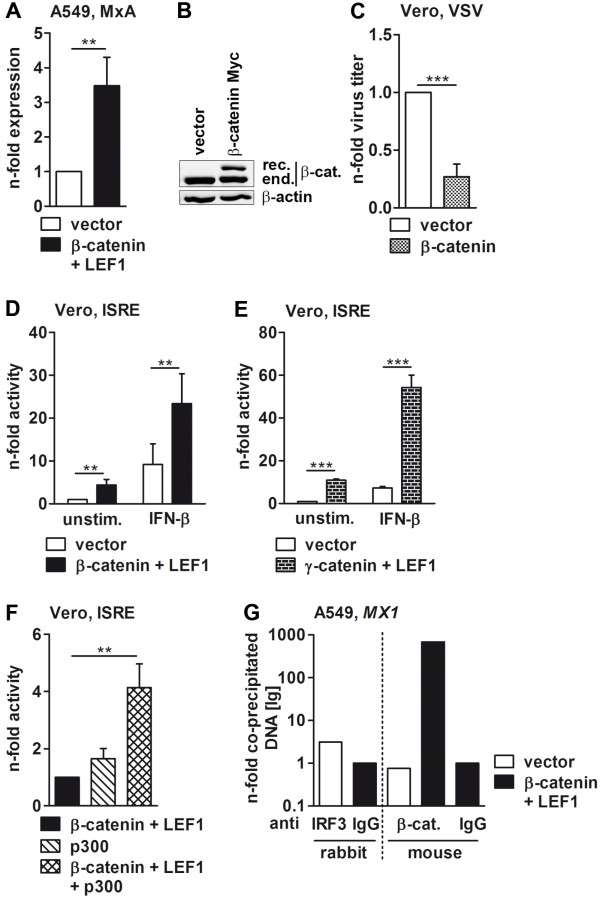
**The ISG promoter activity is triggered by β- and γ-catenin. (A)** A549 cells were transfected with β-catenin and LEF1 for 30 h, and the mRNA level of the type I and type III IFN-dependent *MX1* gene was measured by qRT-PCR. The mRNA amount of empty vector-transfected cells was taken as unity. **(B and C)** Vero cells transfected for 24 h with indicated plasmids were infected with vesicular stomatitis virus (VSV) (MOI = 0.0001) for an additional 24 h. Subsequently, the overexpression of β-catenin was confirmed by immunoblotting of corresponding RIPA lysates **(B)** and the propagation of VSV by standard plaque titration assay **(C)**. **(D and E)** Vero cells were co-transfected with the ISRE luciferase reporter gene and plasmids coding for proteins indicated in column legends. After 24 hours, Vero cells were left unstimulated or treated with 100 U/ml IFN-β for 8 h. The y-axis represents the relative reporter gene activity with luciferase activity of unstimulated, empty vector-transfected cells being set to one. **(F)** Vero cells were transfected with the ISRE luciferase reporter gene, and its activity in β-catenin- and LEF1-overexpressing cells was measured in the presence or absence of co-transfected p300. The luciferase activity of β-catenin and LEF1-transfected cells was arbitrarily taken as unity. **(G)** A549 cells were transfected with empty vector or plasmids coding for β-catenin and LEF1 for 30 h, and the interaction of cellular proteins with the DNA was analyzed by ChIP assays using specific antibodies to IRF3 or β-catenin. The co-immunoprecipitated DNA was amplified by qRT-PCR using specific primers for the promoter region of the *MX1* gene and is given as the n-fold amount to the IgG control. Representative values from one of three repeated experiments are depicted.

The fact that β-catenin positively regulates IFN-β transcription suggests that expression of interferon-induced genes should, in turn, also be upregulated in the presence of the active β-catenin. This was indeed the case. Overexpression of β-catenin and LEF1 efficiently enhanced the synthesis of MxA mRNA in A549 epithelial cells (Figure 5A). The presence of exogenous β-catenin and LEF1 was also confirmed by qRT-PCR (Additional file 1: Table S1). Thus, one might further speculate that suppression of influenza virus replication, as seen in the presence of overexpressed ectopic or upregulated endogenous β-catenin (Figure 1) is based on the induction of IFN-β that exerts antiviral control. To verify this role of β-catenin in antiviral defense, the degradation-resistant mutant of the protein was overexpressed in Vero cells (Figure 5B) that harbor defective type I interferon genes and, therefore, cannot express endogenous IFN-α/β [42]. These cells were subsequently infected with vesicular stomatitis virus (VSV), a pathogen that is highly sensitive to IFN-β [46,47]. VSV is expected to propagate in control vector-transfected and in β-catenin-overexpressing cells with a similar intensity, given the hypothesis is supported. In contrast to the expectation, VSV replicated in β-catenin-overexpressing cells less efficiently than in control cells (Figure 5C), suggesting that β-catenin might, in addition to IFN-β, positively regulate the expression of other proteins with antiviral potential.

Besides type I, also type III IFNs possess antiviral activity [48]. IFN-λ is known to be involved in the induction of MxA expression [49] and was recently discovered as an important antiviral agent that reduced influenza A virus propagation [50]. Thus, one might speculate that β-catenin can enhance the *MX1* transcription shown in Figure 5A indirectly, via induction of IFN-λ. However, a significant increase in IFN-λ mRNA transcription was not detected in β-catenin- and LEF1-overexpressing cells (Additional file 1: Figure S3), suggesting that IFN-λ is not responsible for the β-catenin-mediated induction of MxA mRNA and, consequently, the reduced VSV replication in Vero cells.

As mentioned previously, IFN-β has no intrinsic antiviral activity, but induces the expression of genes that code for proteins with antiviral function via activation of the JAK/STAT pathway. To elucidate whether the β-catenin/LEF1 complex directly influences the transcription of interferon-stimulated genes, Vero cells were transfected with a luciferase reporter gene construct harboring interferon-stimulated response element (ISRE) motifs, and its activity was analyzed in the presence or absence of β-catenin and LEF1. Vero cells were chosen to avoid the effect of endogenous IFN-β molecules on ISRE activity, the induction of which by the transcriptional complex has been shown above. Overexpression of β-catenin and LEF1 simultaneously stimulated ISRE-driven transcription (Figure 5D, left panel). To mimic the cytokine response during virus infection, transfected Vero cells were additionally stimulated with 100 U/ml of recombinant IFN-β (Figure 5D, right panel). This resulted in an increase of reporter gene activity in control cells transfected with empty vector, thus, confirming the functionality of the reporter plasmid. IFN-β stimulation of Vero cells transfected with β-catenin and LEF1 further enhanced the transcription driven by ISRE motifs, but the fold of induction was less to that in unstimulated cells, suggesting that only STAT transcription factors, but not the β-catenin/LEF1 transcription factor, were activated by interferon stimulation. A similar effect on luciferase activation was seen when γ-catenin instead of β-catenin was expressed in Vero cells (Figure 5E), thus, confirming that both catenins have the same antiviral molecular mechanism.

Zhang et al. showed that activated STAT1 recruits the CBP/p300 co-activator to the transcriptional complex that binds to interferon-stimulated gene promoters [51]. Hence, considering the ability of β-catenin to bind CBP/p300 as well, we asked whether these factors regulate the transcription of ISRE in a cooperative manner. Co-expression of p300, in addition to β-catenin and LEF1, clearly showed an additive effect on the ISRE reporter gene activity in Vero cells. The luciferase activity was four times higher than in cells expressing β-catenin and LEF1 alone (Figure 5F). Thus, similar to IRF3-driven transcription of the *IFNB1* gene, the β-catenin/LEF1 transcriptional complex supports the transcription of interferon-stimulated genes in concert with the general transcriptional co-activator p300.

To verify whether the effect of β-catenin on transcriptional activity of ISRE promoters is induced by binding to the promoter region, similar to that observed for the IFN-β promoter, we analyzed if β-catenin can interact with the promoter region of ISGs by ChIP assays. Again, immunoprecipitation of IRF3, which is known to bind the ISRE motif of ISGs [52,53], was used as positive control. As expected, IRF3 immunoprecipitates were positive for *MX1* promoter DNA, as detected by qRT-PCR, but more reasonable, the β-catenin immunoprecipitates were also positive for *MX1* promoter DNA, although only after overexpression of both β-catenin and LEF1 (Figure 5G).

In conclusion, the β-catenin-induced enhancement of ISRE promoter activity is mediated by binding of β-catenin to ISRE promoter DNA, similar to the IFN-β-induced transcription.

### Influenza A virus inhibits the β-catenin-mediated transcriptional activation of LEF/TCF-dependent genes

**Figure 6:**
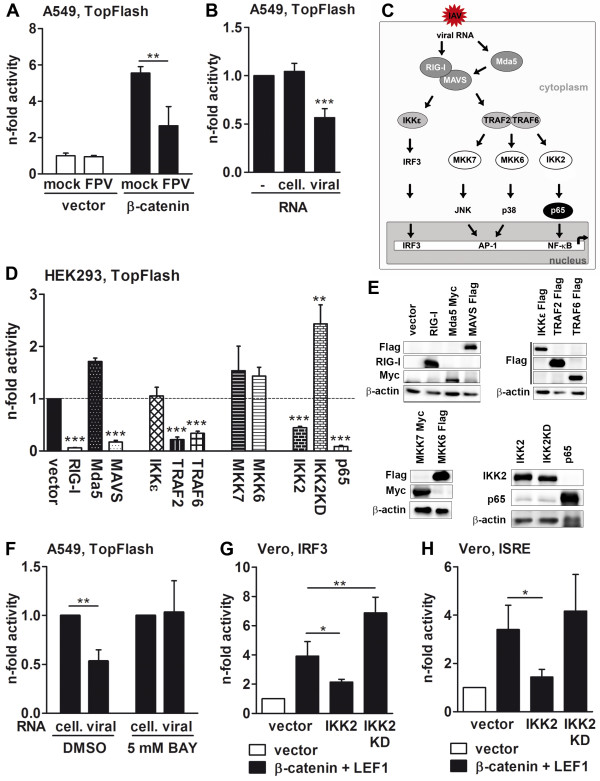
**IAV infection inhibits β-catenin-mediated transcriptional activation of LEF/TCF-dependent target genes. (A and B)** A549 cells were transfected with the TopFlash reporter construct together with an empty vector or a plasmid coding for β-catenin. At 24 h post transfection, the cells were infected with FPV (MOI = 5) or stimulated (via transfection) for an additional 8 h with 500 ng of cellular or viral RNA or left untreated. Subsequently, the promoter activity was measured. The luciferase activity of mock-infected or unstimulated but empty vector-transfected cells was taken as unity. **(C)** A schedule of signaling cascades activated by IAV RNA is depicted (adapted from [10,55,56]). **(D and E)** HEK293 cells were transfected with the TopFlash reporter gene construct together with β-catenin and plasmids coding for the indicated proteins. At 30 h post transfection, the promoter activity was measured **(D)**. The luciferase activity of β-catenin and empty vector-transfected cells was arbitrarily taken as unity. Overexpression of the recombinant proteins was analyzed by Western blotting. The β-actin immunoblots always served as loading controls **(E)**. **(F)** A549 cells transfected with the TopFlash reporter plasmid together with β-catenin were stimulated with 5 mM of BAY inhibitor or DMSO as control. At 16 h post stimulation, cells were transfected with 500 ng cellular or viral RNA for an additional 8 h and the promoter activity was measured. The luciferase activity of cells stimulated with cellular RNA was always taken as unity. **(G and H)** Vero cells were transfected with reporter gene plasmids containing either the IRF3 responsive elements of the IFN-β enhanceosome **(G)** or the ISRE motif **(H)** along with plasmids coding for proteins indicated in column legends. The promoter activity was measured 30 h post transfection. Luciferase activity of cells transfected with any vector control was arbitrarily taken as unity.

Regulation of gene transcription is a well-known function of β-catenin. To test whether IAV infection influences this function, A549 cells were transiently transfected with the catenin-LEF/TCF-dependent TopFlash reporter construct harboring LEF/TCF binding sites upstream of the thymidine kinase minimal promoter together with a plasmid encoding the phosphorylation-refractory β-catenin mutant. Subsequently, the cells were infected with the avian FPV strain (Figure 6A). As anticipated, the stabilized β-catenin protein strongly activated the transcription of the reporter gene. The effect was specific, as transfection of the FopFlash reporter vector containing mutated LEF/TCF binding sites was not activated by β-catenin (Additional file 1: Figure S4). However, in contrast to our expectation, IAV infection strongly repressed the β-catenin-dependent activation of the reporter gene (Figure 6A). Similar results were obtained when the human PR8 isolate was used (Additional file 1: Figure S5), indicating that the observed effect is not virus strain specific. Furthermore, accumulation of viral but not cellular RNA, which was transfected into A549 cells, was sufficient for repression of the β-catenin-dependent transcription (Figure 6B).

Influenza RNA molecules are sensed by cytoplasmic helicases, the RIG-I and melanoma differentiation-associated gene 5 (Mda5) [54], resulting in activation of several cellular signaling cascades that preferentially funnel into the transcriptional induction of the *IFNB1* gene (Figure 6C) [10,55,56]. To identify the signaling cascades responsible for inactivation of the TopFlash reporter gene, the β-catenin-dependent activation of the promoter was analyzed in cells that also overexpress proteins representing different members of viral RNA-induced signaling pathways in addition to β-catenin. The results presented in Figure 6D clearly demonstrate that RIG-I- but not Mda5-mediated signaling was responsible for inhibition of the TopFlash reporter gene, as overexpression of active RIG-I or its downstream effector MAVS abolished β-catenin-induced promoter activity. Walking down the RIG-I/MAVS-dependent signaling cascades revealed that activation of the IKK2-p65 axis, but not of IRF3 or JNK and p38 branches, efficiently inhibited TopFlash reporter gene activity (Figure 6D). Western blot images shown in Figure 6E demonstrate the efficient overexpression of different molecules used for analysis of signaling pathways responsible for suppression of β-catenin function. Furthermore, treatment of cells with the IKK2-specific BAY inhibitor [57] prior to stimulation with viral RNA confirmed the involvement of the NF-κB cascade in β-catenin inactivation, as cells stimulated with the BAY inhibitor were unable to downregulate the TopFlash promoter activity (Figure 6F).

The NF-κB pathway is obligatorily activated after viral infection [58]. Given that activation of this pathway inhibits the transcription of LEF/TCF-dependent target genes, the β-catenin-mediated transcriptional support of the IFN-β and ISRE-dependent genes should be inhibited on viral infection as well. To test this assumption, the luciferase activity of IRF3- and ISRE-dependent reporter genes was analyzed in the presence of an active or inactive IKK2 kinase. Results presented in Figures 6G and H show that both IRF3- and ISRE-dependent transcription were efficiently inhibited by overexpression of a functionally active IKK2 kinase, whereas the dominant-negative form of the IKK2 had rather an opposite or no effect.

Thus, the results presented here show that β-catenin supports the transcription of genes involved in regulation of the innate immune response to IAV infections, on the one hand, and that the functional activity of the β-catenin is at the same time suppressed by the IAV-mediated activation of the NF-κB pathway, on the other hand.

## Discussion

Transcription and translation of viral RNA and proteins are strictly dependent on the host cell machinery [59]. Different cellular signaling cascades are known to be activated upon viral infection, like the MAP kinases ERK1/2, JNK and p38 or the classical NF-кB pathway, which support pro- or antiviral actions [34,60,61]. Furthermore, there are several cellular molecules identified that directly mediate antiviral activity, like different ISG proteins [12].

Ongoing interaction studies between influenza A virus proteins and cellular factors [62] as well as siRNA screens [63] suggest that Wnt signaling does not only control processes like cell differentiation, communication, apoptosis/survival and proliferation but also plays a role during virus infections. In this study, the role of β-catenin, the intracellular content of which can be regulated for example by different factors including Wnt proteins, was elucidated in IAV-infected cells. We demonstrate here that β-catenin is involved in IAV replication and that its accumulation is sufficient to reduce virus propagation. Stimulation of human lung epithelial cells with either natural (Wnt3a) or artificial (LiCl) agents that inactivate the GSK-3α and β kinases [36] and, thus, induce the cellular accumulation of β-catenin, reduced IAV replication. These results are consistent with the data presented by Kumar et al. where LiCl stimulation reduced HIV progression in peripheral blood mononuclear cells [64] and show the potential role of the canonical Wnt-signaling in anti-influenza response.

It is well known that β-catenin plays a dual role within the cell. On the one hand, it is part of adherens junctions at the cell membrane and, on the other hand, it interacts with LEF/TCF and acts as a transcription factor [19]. Due to the fact that the transcriptionally active β-catenin reduces viral propagation, we assumed that the effect is caused at the transcriptional level and not by mediating the association of viral RNPs to the actin cytoskeleton, like it was previously shown for parainfluenza virus infections [65]. The first and most potent wave of cellular innate immune response to IAV infection is mainly regulated at the transcriptional level. Here, we have shown that the activity of the IFN-β enhanceosome is modulated by the β-catenin/LEF1 protein complex. The increased promoter activity was mediated via the IRF3-dependent PRDIII-I region but not by enhanced homodimerization of the interferon regulating factor 3. Thus, the β-catenin/LEF1 complex does not act within the viral RNA-mediated IRF3 activation pathway but by direct interaction with the transcriptional complex. Recently Yang et al. showed that β-catenin can bind to IRF3 in HEK293 cells [44], and here we showed that the presence of the transcriptional co-factor p300 significantly raised the β-catenin-induced, IRF3-dependent promoter activity. The association of the general transcriptional co-factor CBP/p300 with β-catenin or IRF3 was demonstrated in several independent studies, as well as the increase of β-catenin or IRF3 target gene transcription by recruiting CBP/p300 [43,45,66-68]. The results of our work further support and enhance these data by showing that the combined action of all three proteins is required for full activation of the IFN-β promoter.

In addition to the enhancement of IFN-β expression, we show here that β-catenin and LEF1 also activate genes of the IFN induced wave of cellular innate immune response to influenza virus infection, namely the transcription of interferon-stimulated genes, whose products directly inhibit transcription and translation of viral RNA and proteins. The fact that the β-catenin/LEF1 protein complex regulates both IFN-β and ISG promoter activity shows that it possess a biphasic antiviral action. In both cases, β-catenin exercised its activity by binding to appropriate DNA promoter regions. This binding is most likely indirect, as β-catenin itself has no DNA-binding domains and there are no classical LEF/TCF binding sites [69] in the promoter regions of either *IFNB1* or *MX1* gene (Additional file 2: Figure S6). It is conceivable that IRF3 is the primary DNA-binding protein and the transcriptional co-activator p300 is the common link between IRF3 and β-catenin/LEF1 protein complex, as co-expression of p300 significantly enhanced the β-catenin/LEF1-mediated transcriptional activity of both IFN-β and ISG promoters.

Cellular γ-catenin is the close homologue of β-catenin and also possesses a dual function, controlling cell-cell adhesion at the membrane [31] and gene transcription in the nucleus [32]. Both catenins bind to cadherin receptors and regulate the activity of common target genes in concert with LEF1 as transcriptional complex [32,33]. Our results provide further functional significance for β- and γ-catenin and underscore their redundancy. Indeed, knockdown of β-catenin by specific RNAi resulted in prompt enhancement of γ-catenin expression, and we showed for the first time that both catenins efficiently enhanced the cellular innate immune response to influenza A viruses by promoting IFN-β and ISG expression.

Recently, it was demonstrated that GSK-3β is inactivated upon influenza A virus infection through phosphorylation at Ser9 by kinases via the phosphatidylinositol 3-kinase (PI3K)/Akt pathway [30]. Here, we showed that β-catenin accumulates in the nucleus after IAV infection. These data suggest that the β-catenin signaling is influenced by virus infection. Lately, Zhu et al. [70] showed that during Sendai virus infections, β-catenin is deacetylated by PKC-activated HDAC6, which results in nuclear translocation of β-catenin and enhanced IRF3-dependent expression of interferon responsive genes. We showed here that β-catenin supports the IRF3-dependent transcription of genes responsible for the cellular innate immune response, the IFN-β and ISGs. In contrast to these results, Baril and co-workers reported that Wnt signaling does not support interferon induction after Sendai virus infection and that Wnt9B and Wnt2B but not the Wnt3a efficiently reduces IFN expression [71]. Although the reason for the discrepancy between these data and results discussed above is not clear yet, different posttranslational modifications of the β-catenin protein due to the different Wnt stimuli as well as to different viruses and cell types used might be a plausible explanation.

Altogether, these results indicate that β-catenin is involved in the cellular defense against different virus types, although in a different way, and, therefore, represents an important antiviral molecule. Interestingly, influenza viruses counteract the antiviral potency of β-catenin by inhibiting its transcriptional activity. While in the case of human cytomegalovirus infection, β-catenin inhibition was mediated by newly synthesized viral proteins [72], the IAV inhibition of β-catenin was induced through accumulation of viral RNA and subsequent activation of the NF-κB signaling cascade. Though the exact molecular mechanism of the p65-induced inhibition of β-catenin-dependent transcription is still unknown, it is very likely that binding of the NF-κB p65 transcription factor to β-catenin is responsible for the inhibition of the latter, as the association of p65 with β-catenin [73-75] and the inhibitory effect of the protein complex onto the TopFlash activity has been reported previously [74-76]. Whether the β-catenin-p65 interaction also regulates the NF-кB activity is still controversy discussed in the literature [73,75,76], nonetheless, no significant differences in NF-κB promoter activity on overexpression of β-catenin and LEF1 were observed in this study.

## Conclusion

We described in this study the anti-influenza potential of the transcriptionally active β- and γ-catenin. We showed that they comprise an antiviral activity by a direct support of (i) the transcription of the *INFB1* gene, the first wave of the cellular innate immune response and (ii) the transcription of the interferon-stimulated genes. However, upon IAV infection, β-catenin-dependent transcription is inhibited via the RIG-I/NF-кB signaling cascade that is activated by accumulated viral RNA.

## Materials and methods

### Cell culture and influenza A virus infection

The human alveolar epithelial (A549), colorectal cancer (SW480), embryonic kidney (HEK293) and green monkey epithelial (Vero) cells were grown in Dulbecco’s minimal essential medium (D-MEM) and Madin-Darby canine kidney (MDCKII) cells in minimal essential medium (MEM) supplemented with 10% bovine fetal serum (Biochrome).

The human influenza virus strain A/Puerto Rico/8/34 (PR8, H1N1) was originally obtained from T. Wolff (Robert-Koch Institute, Berlin, Germany), and the avian influenza virus strain A/FPV/Bratislava/79 (FPV, H7N7) was used with kind permission of S. Pleschka (Institute of Virology, Giessen, Germany); both were propagated in MDCKII cells. The vesicular stomatitis virus strain Indiana (VSV) was a gift from T. Wolff (Robert-Koch Institute, Berlin, Germany) and was propagated in Vero cells. For infection, cells were washed with PBS and incubated with viruses diluted in PBS containing 0.2% bovine albumin, 100 U/ml penicillin and 0.1 mg/ml streptomycin (PAA) for 30 min at 37°C with indicated MOIs (multiplicity of infection) or without virus particles (mock infection) as a control. After washing, the virus solution was replaced with growth medium containing 0.2% bovine albumin and antibiotics, and cells were incubated for the indicated times. To quantify virus propagation, cell supernatants were taken 24 h post infection and analyzed in standard plaque titration assays as described previously [77]. Recombinant human IFN-β was purchased from PBL Interferon Source, BAY11-7085 from Sigma-Aldrich, recombinant human Wnt3a from R&D Systems and lithium chloride (LiCl) was obtained from Carl Roth GmbH.

### CHIP analysis

For analysis of protein-DNA interactions, the Magna ChIP A Kit purchased from Millipore was used according to the manufacturer’s instructions. For each reaction, 5 μg of a specific antibody or the control serum were utilized. Immunoprecipitated DNA was quantified by qRT-PCR with primers specific for the promoter region of the investigated gene. The amount of immunoprecipitated DNA in each sample was calculated in comparison to the total DNA per sample. Each yielded value was then normalized to IgG controls. The primers were:

*IFNB1* fw 5′-GAATAGGAAAACTGAAAGGGAGA-3′,

*IFNB1* rev 5′ -GTGTCGCCTACTACCTGTTGTG-3′;

*MX1* fw 5′-CCCCTGGATTCTGAAGTCTGA-3′,

*MX1* rev 5′-TTTCCCGGACAATTCAGTTTC-3′.

### pppRNA generation, RNA isolation and quantitative real time-PCR (qRT-PCR)

The 5′-triphosphate modified RNA (pppRNA) was generated as described previously [78]. For production of viral RNA, A549 cells were either mock- or PR8-infected with an MOI of 5. Then, 8 h post infection the total RNA was isolated using the RNeasy Kit (Qiagen). RNA isolated from mock-infected cells was used as a control and referred to as cellular RNA, while RNA isolated from IAV-infected cells was termed viral RNA.

For synthesis of cDNA, 1 μg of total RNA, isolated from cells using the RNeasy kit (Qiagen), were reverse-transcribed with RevertAID polymerase (Fermentas) according to the manufacturer’s instructions.

The qRT-PCR analysis of obtained cDNA probes was performed with LightCycler 480 (Roche) and 2× SYBR Green Brilliant III Master Mix (Agilent Technologies/Stratagene). For that, 0.5 μl of synthesized cDNA was mixed with 4 μl of 2× SYBR Green Brilliant Master Mix and 0.6 μl of each primer [10 pmol/μl] and brought up to a total volume of 12 μl with RNase free water. The following primer pairs were used for mRNA analysis: human GAPDH: 5′-GCAAATTTCCATGGCACCGT-3′ and 5′-GCCCCACTTGATTTTGGAGG-3′; human MxA: 5′-GTTTCCGAAGTGGACATCGCA-3′ and 5′-GAAGGGCAACTCCTGACAGT-3′; human IFN-λ: 5′-GTGCTGGTGACTTTGGTGCTA-3′ and 5′-GAGAAGCCTCAGGTCCCAAT-3′; mouse β-catenin: 5′-ATGGCTTGGAATGAGACTGC-3′ and 5′-CTCCATCATAGGGTCCATCC-3′; mouse LEF1: 5′-CCCGTCAGATGTCAACTCCA-3′ and 5′-CGTGATGGGATAAACAGGCT-3′. mRNA amounts were normalized to GAPDH, and relative changes in expression levels (n-fold) were calculated according to the 2^-ΔΔCT^ method [79].

### Transfection and reporter gene assays

For siRNA-based knockdown, 5 × 10^5^ SW480 cells were transfected in suspension with 150 ng of β-catenin siRNA (5′-CTCGGGATGTTCACAACCGAA-3′) or control siRNA (5′-UUCUCCGAACGUGUCACGU-3′) (Qiagen) using the HiPerfect (Qiagen) transfection reagent. For analysis of mRNA expression, A549 cells (5 × 10^5^ per 6-well dish) were transfected with X-treme Gene HP (Roche) following the manufacturer’s instructions. For reporter gene assays, A549 or Vero cells (2 × 10^5^ or 1.5 × 10^5^ per 12-well dish) were transfected with various combinations of protein-expressing plasmids along with reporter gene constructs using Lipofectamine 2000 (Invitrogen). HEK293 cells (2 × 10^5^ per 12-well dish) were transfected with polyethylenimine (PEI) in a 1:3 ratio (1 μg DNA: 3 μl PEI [1 mg/ml]). Typically 0.3 μg of luciferase reporter plasmid DNA was co-transfected with 0.5 μg of each expression plasmid encoding either empty vector or indicated proteins which were: pEGFP RIG-I Card (gift of F. Weber, University Hospital Freiburg, Germany), pcDNA3 Flag MAVS [80], pEF1 Myc-His Mda5 (gift of T. Wolff, Robert-Koch Institute, Berlin, Germany), pEFP Flag IKKϵ, pRK Flag TRAF2, pFlag TRAF6, pcDNA3 MKK6 Flag (given by R. Davis, Medical School, UMass), pCS3 + MT MKK7 (gift of M. Kracht, Rudolf-Buchheim-Institute of Pharmacology, University of Giessen, Germany), pEGFP IRF3DD, pcDNA3 IKK2 and pcDNA3 IKK2KD (plasmid collection of the IMV, Münster, Germany), pcDNA3 β-catenin S33A 6xMyc (murine) and pCG murine LEF1 HA (murine) [35], pFCΔ MEKK1 [81] or pCMV p300 HA (given by K.-H. Klempnauer; Institute of Biochemistry, Münster, Germany). The pcDNA3 p65 expression plasmid was generated via cloning the PCR amplicon of pGal4 p65 [82] into BamHI/NotI restriction sites of the pcDNA3.1 vector. The pcDNA3 6xMyc γ-catenin expression plasmid was obtained by recloning the plakoglobin cDNA from the pGAD424 plakoglobin [35] plasmid into the pcDNA3 6xMyc vector.

The used luciferase reporter gene constructs were described previously: pTATA IFNβ luc, pTATA-4xIRF3 luc and NF-кB-driven pGL3-5xNF-kB luc [78], AP1–driven pB4xAP1/Etsluc reporter gene construct [81], pTA ISRE luc (Clontech Laboratories), pTK-TopFlash and pTK-FopFlash [35]. Luciferase activities were measured 30 h post transfection using the luciferase protocol as described in [83] and the MicroLumatPlus LB 96 V luminometer (Berthold Technologies). The relative light units (RLUs) were normalized to protein concentrations, determined using the Bradford dye [84], and given as the n-fold activity of the indicated control.

Total amounts of transfected DNA per cell dish varied depending on the experimental protocol, but the total DNA content per dish within the experiment was kept constant by adding the appropriate amount of empty expression vector. Each transfection was carried out in duplicate or triplicate.

### Protein lysates and Western blot analysis

Cells were washed with PBS and lysed in RIPA buffer (25 mM Tris/HCl pH 8, 137 mM NaCl, 10% glycerol, 0.1% SDS, 0.5% DOC, 1% NP-40, 2 mM EDTA, 1 mM sodium vanadate, 5 μg/ml leupeptin, 5 μg/ml aprotinin and 200 μM pefablock). The lysates were cleared by centrifugation at 10,000 g for 10 min at 4°C. Afterwards, the protein amount was determined by BCA (bicinchoninic acid) protein reagent assay (Thermo Fisher Scientific), and 20 μg of total protein lysates was resolved by discontinuous SDS-PAGE. To analyze the IRF3 dimerization, a native PAGE was performed as described previously [85]. Briefly, 2 × 10^6^ cells were scraped into 50 μl of lysis buffer (50 nM Tris–HCl pH 8, 1% NP-40, 150 mM NaCl, 100 μg/ml leupeptin and 5 mM sodium vanadate), and supernatants were cleared by centrifugation in a standard table-top centrifuge at maximum speed at 4°C. Equal protein amounts in sample buffer (125 nM Tris–HCl pH 8, 30% glycerol, bromphenol blue) were separated on a 7.5% polyacrylamide gel with a two-buffer system (upper chamber buffer: 25 mM Tris–HCl pH 8.4, 192 mM glycine, and 1% sodium deoxycholate; lower chamber buffer: 25 mM Tris–HCl pH 8.4 and 192 mM glycine) at constant 20 mA on ice. Before blotting of proteins onto nitrocellulose membranes, the gel was soaked in SDS-PAGE running buffer (25 mM Tris–HCl pH 8.4, 250 mM glycine and 0.1% SDS) for 30 min. After electroblotting onto nitrocellulose membranes, the specific proteins were detected by Western blot analysis with appropriate antibodies using the ECL detection system. The used antibodies were: mAb anti-β-catenin clone 14 and mAb anti-γ–catenin (BD Transduction Laboratories), mAb anti-HA (clone 12CA5) and mAb anti-myc (clone 9E10) (ATCC Cell Biology Collection), pAb anti-influenza PB1 VK20, mAb anti-influenza NS1 clone NS1 23–1, pAb anti-IKKα/β and pAb anti-IRF3 (Santa Cruz Biotechnology), mAb anti-p65, mAb anti-GSK-3β and pAb anti-p-GSK-3β Ser9 (Cell Signaling), mAb anti-RIG-I (Enzo Life Sciences), mAb anti-Lamin C (Abcam) mAb anti-Flag, mAb anti-β-actin and mAb anti-α-tubulin (Sigma-Aldrich). Antiserum against viral PB2 protein was a kind gift from Dr. E. Fodor (Sir William Dunn School of Pathology, Oxford, UK [86]) The secondary antibodies were obtained from Jackson Immunoresearch (donkey anti-goat IgG-POX and goat anti-mouse IgG-POX) or Biorad technologies (goat anti-rabbit IgG-HRP).

Preparation of cytosolic and nuclear fractions was performed as described in [87]. Briefly, 2.5 × 10^6^ HEK293 cells were treated as indicated in the figure legend, washed twice with ice-cold PBS, scrapped off and harvested by centrifugation (5 min 650 g at 4°C). Cell pellets were lysed in 1 ml Roeder A buffer (10 mM HEPES pH 7.9, 1.5 mM MgCl_2_, 10 mM KCl supplemented with 0.5 mM DTT, 1 mM sodium vanadate, 0.2 mM pefablock, 5 mg/ml leupeptin, and 5 mg/ml aprotinin) and incubated on ice for 10 min. Then, NP-40 was added to a final concentration of 0.3% and cell lysates were mixed and incubated for an additional 10 min on ice. Next, nuclei were sedimented by centrifugation (10 min at 2650 g and 4°C) and the supernatant was collected (referred as cytoplasmic fraction). The pellet was washed with 1 ml Roeder A buffer and subsequently resuspended in 300 μl of Roeder C buffer (25% (v/v) glycerol, 0.3 M NaCl, 1.5 mM MgCl_2_, 20 mM HEPES pH 7.9 supplemented with 0.5 mM DTT, 1 mM sodium vanadate, 0.2 mM pefablock, 5 mg/ml leupeptin, and 5 mg/ml aprotinin). After incubation for over-night in an overhead rotator at 4°C the nuclear fraction was clarified by centrifugation (30 min, 20000 g at 4°C) and used for SDS-PAGE and Western blotting.

### Software

For detection of signals, quantification, evaluation or illustration of the results, the following software was used: XStella_2.14, WinGlow-Control Programm LB96V, Aida Image Analyser v.4.21, Excel (Microsoft office 2010), Adobe Photoshop CS3 and GraphPad Prism 5.

### Statistical analysis

Statistical significance between samples was determined using the homoscedastic Student’s *t*-test in two-tailed distribution. Values of *p ≤ 0.05, **p ≤ 0.01 and ***p ≤ 0.001 are indicated. If no specification is denoted in the legends, mean values ± SD from at least three independent experiments are depicted. Some figures display mean values from one representative out of three independent experiments. This is due to variations in the basal promoter activity or viral propagation between the different repeats while the findings are comparable. In these cases, mean values ± SD are calculated from three biological replicates.

## Abbreviations

AP-1: Activator protein-1; CBP: CREB binding protein; FPV: Fowl plaque virus A/FPV/Bratislava/79 (H7N7); GSK: Glycogen synthase kinase; IAV: Influenza A virus; IFN: Interferon; IKK: I-kappa-B kinase; IRF: Interferon regulatory factor; ISG: Interferon-stimulated gene; ISRE: Interferon-stimulated response element; LEF: Lymphoid enhancer factor; LiCl: Lithium chloride; MxA: Myxovirus resistance gene A; MAVS: Mitochondrial antiviral-signaling protein; MOI: Multiplicity of infection; NF-κB: Nuclear factor kappa-light-chain-enhancer of activated B-cells; p.i: Post infection; PR8: A/Puerto Rico/8/34 (H1N1); RIG: Retinoic acid inducible gene; STAT: Signal transducers and activators of transcription; TBK-1: TANK-binding kinase 1; TCF: T-cell factor; VSV: Vesicular stomatitis virus.

## Competing interests

The authors declare that they have no competing interests.

## Authors’ contributions

AH performed experiments, analyzed the data and designed the Figures. YB and CN were also involved in performing experiments and assisted with analyzing and interpreting data. AH, SL and VW designed the research, analyzed the data and wrote the manuscript. All authors read and approved the final manuscript.

## Supplementary Material

Additional file 1: Figure S1 IAV infection results in phosphorylation of GSK-3β. (A and B) A549 cells were infected with influenza A/FPV/Bratislava/79 (FPV, H7N7) or influenza A/Puerto Rico/8/34 (H1N1) (MOI = 5) for indicated times. RIPA lysates were analyzed for designated proteins. Infection was verified by PB1 and NS1 and equal protein loads by GSK-3β and β-actin. **Figure S2.** IRF3 dimerization is independent on β-catenin and LEF1 overexpression. A549 cells overexpressing β-catenin and/or LEF1 were stimulated with 1 μg of cellular or viral RNA for 4 h and IRF3 dimerization was analyzed by non-denaturing electrophoresis and Western blotting. Equal protein loads were verified by β-actin immunoblotting. **Figure S3.** Analysis of IFN-λ mRNA expression in β-catenin- and LEF1-overexpressing cells. A549 cells were transfected with β-catenin and LEF1 for 30 h, and the mRNA level of the *IFNL1* gene was measured by qRT-PCR. **Figure S4.** Comparison of FopFlash and TopFlash promoter activity. A549 cells were transfected with indicated reporter constructs together with the empty vector or β-catenin. 24 h later, cells were stimulated with 500 ng of cellular or viral RNA for an additional 8 h and the promoter activity was measured. The luciferase activity of cellular RNA-stimulated and with empty vector-transfected cells was taken as unity. A representative image out of three independent experiments is shown. **Figure S5.** Regulation of β-catenin-dependent transcription upon PR8 infection. A549 cells were transfected with the TopFlash reporter construct and empty vector or a plasmid encoding β-catenin. 24 h post transfection, the cells were infected with PR8 (MOI = 5) for an additional 8 h, and the promoter activity was measured. A representative image out of three independent experiments is shown. **Table S1.** Detection of recombinant β-catenin and LEF1 by qRT-PCR after transient transfection of A549 cells. The human GAPDH mRNA level was used as control. Click here for file

Additional file 2: Figure S6 Overview of *IFNB1* or *MX1* promoter regions with binding sites of potential transcription factors. Sequences of *IFNB1* (chr9:21077842–21078441) (A) and *MX1* (chr21:42791953–42792552) (B) promoter regions that have been amplified for ChIP assay analysis are shown. The potential transcription factors and their binding sites were verified using the bioinformatics tool NSITE of Softberry Inc. Nucleotides are numbered beginning at transcription sites. The arrows denote the direction of transcription. Click here for file
