# Supplementary material for: β-catenin promotes the type I IFN synthesis and the IFN-dependent signaling response but is suppressed by influenza A virus-induced RIG-I/NF-κB signaling
Source: Cell Commun Signal. 2014 Apr 26;12:29. doi: 10.1186/1478-811X-12-29 (PMC4021428; doi:10.1186/1478-811X-12-29)
Supplement: Additional file 1: Figure S1 — IAV infection results in phosphorylation of GSK-3β. (A and B) A549 cells were infected with influenza A/FPV/Bratislava/79 (FPV, H7N7) or influenza A/Puerto Rico/8/34 (H1N1) (MOI = 5) for indicated times. RIPA lysates were analyzed for designated proteins. Infection was verified by PB1 and NS1 and equal protein loads by GSK-3β and β-actin. Figure S2. IRF3 dimerization is independent on β-catenin and LEF1 overexpression. A549 cells overexpressing β-catenin and/or LEF1 were stimulated with 1 μg of cellular or viral RNA for 4 h and IRF3 dimerization was analyzed by non-denaturing electrophoresis and Western blotting. Equal protein loads were verified by β-actin immunoblotting. Figure S3. Analysis of IFN-λ mRNA expression in β-catenin- and LEF1-overexpressing cells. A549 cells were transfected with β-catenin and LEF1 for 30 h, and the mRNA level of the IFNL1 gene was measured by qRT-PCR. Figure S4. Comparison of FopFlash and TopFlash promoter activity. A549 cells were transfected with indicated reporter constructs together with the empty vector or β-catenin. 24 h later, cells were stimulated with 500 ng of cellular or viral RNA for an additional 8 h and the promoter activity was measured. The luciferase activity of cellular RNA-stimulated and with empty vector-transfected cells was taken as unity. A representative image out of three independent experiments is shown. Figure S5. Regulation of β-catenin-dependent transcription upon PR8 infection. A549 cells were transfected with the TopFlash reporter construct and empty vector or a plasmid encoding β-catenin. 24 h post transfection, the cells were infected with PR8 (MOI = 5) for an additional 8 h, and the promoter activity was measured. A representative image out of three independent experiments is shown. Table S1. Detection of recombinant β-catenin and LEF1 by qRT-PCR after transient transfection of A549 cells. The human GAPDH mRNA level was used as control. [file 1478-811X-12-29-S1.pdf]

Figure S1

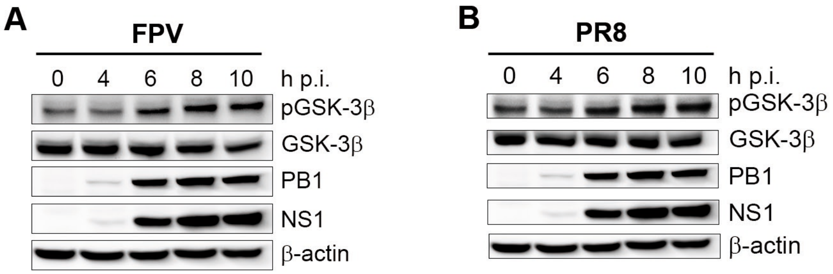

Figure S2

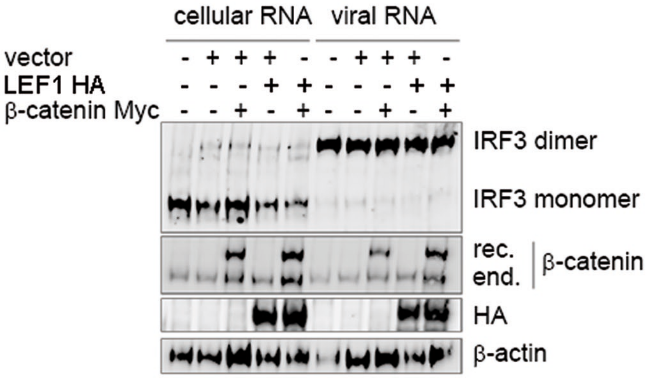

Figure S3

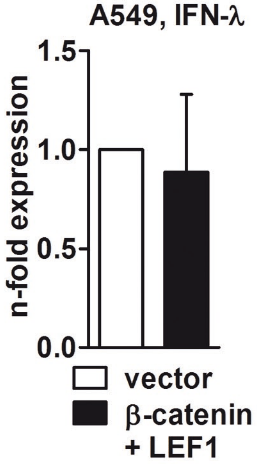

Figure S4

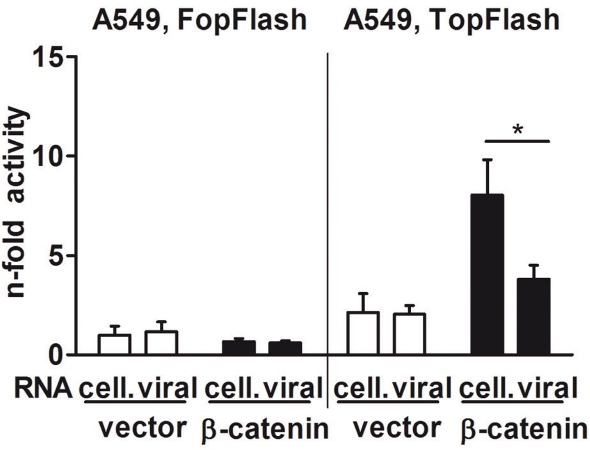

Figure S5

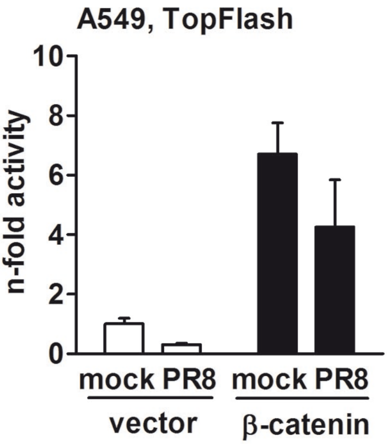

Table S1

	vector			β-catenin + LEF1		
Primer	GAPDH	β-catenin	LEF1	GAPDH	β-catenin	LEF1
Cp value	14.75	32.28	35.00	14.66	17.04	11.96
ΔCp	-	18.56	20.72	-	2.23	-2.49
