# Supplementary figures and images for: β-catenin promotes the type I IFN synthesis and the IFN-dependent signaling response but is suppressed by influenza A virus-induced RIG-I/NF-κB signaling
Source: Cell Commun Signal. 2014 Apr 26;12:29. doi: 10.1186/1478-811X-12-29 (PMC4021428; doi:10.1186/1478-811X-12-29)

Figure S6

A

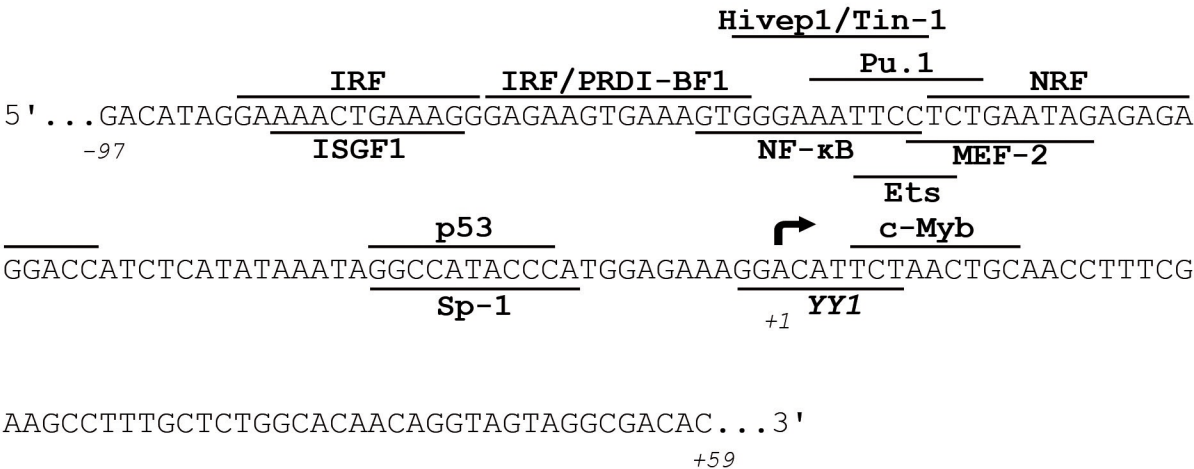

B

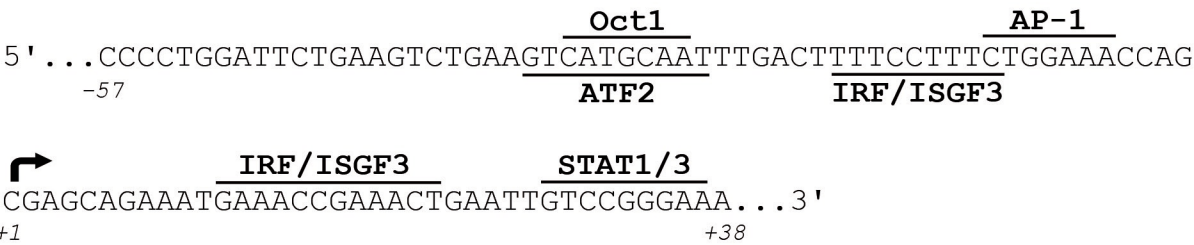

Supplement: Additional file 2: Figure S6 — Overview of IFNB1 or MX1 promoter regions with binding sites of potential transcription factors. Sequences of IFNB1 (chr9:21077842–21078441) (A) and MX1 (chr21:42791953–42792552) (B) promoter regions that have been amplified for ChIP assay analysis are shown. The potential transcription factors and their binding sites were verified using the bioinformatics tool NSITE of Softberry Inc. Nucleotides are numbered beginning at transcription sites. The arrows denote the direction of transcription. [file 1478-811X-12-29-S2.pdf]
